# EPIDEMIOLOGICAL PROFILE AND WAITING TIME FOR OSTEOSYNTHESIS OF TRANSTROCHANTERIC FRACTURES

**DOI:** 10.1590/1413-785220253305e290192

**Published:** 2025-09-22

**Authors:** Felipe da Silva de Melo, Lara Letícia Brito de Andrade, Gabriel Vale do Monte Sobreira, William Roberto Paredes Argotte

**Affiliations:** 1 Fundação de Ensino e Pesquisa em Ciencias da Saude (FEPECS), Brasilia, DF, Brazil.; 2 Secretaria de Saude do Distrito Federal, Ortopedia e Traumatologia, Brasilia, DF, Brazil.

**Keywords:** Femoral Fractures, Osteosynthesis, Fracture, Indicators of Morbidity and Mortality, Treatment Delay, Aged, Rehabilitation, Fraturas do Fêmur, Osteossíntese, Morbimortalidade, Atraso no Tratamento, Idoso, Reabilitação

## Abstract

**Objective:**

To evaluate the epidemiological profile and waiting time for osteosynthesis in patients admitted due to intertrochanteric fracture in a public hospital in the Federal District of Brazil.

**Methods:**

This observational, descriptive, and retrospective study involved patients over 18 years old with intertrochanteric fractures between June and December 2023. Demographic, clinical data, and information about waiting time for osteosynthesis were collected. Analyses were performed using descriptive statistics and logistic regression.

**Results:**

The majority of patients (61.4%) were female, with a mean age of 74 years. The average time between fracture and care was 3.4 days, and from fracture to osteosynthesis was 22.7 days. The mortality rate was 6.8%, with higher prevalence in elderly women with comorbidities.

**Conclusions:**

The study demonstrates that a prolonged time between fracture and osteosynthesis is associated with worse clinical outcomes, particularly in elderly patients with comorbidities. Optimizing the waiting time for surgery is essential to reduce morbidity and mortality and improve patient recovery. ***Level of Evidence IV; Observational, Descriptive, and Retrospective Study.***

## RESUMO

**Objetivo:**

Avaliar o perfil epidemiológico e o tempo de espera para a osteossíntese de pacientes internados devido à fratura transtrocanteriana em um hospital público do Distrito Federal.

**Métodos:**

Estudo observacional, descritivo e retrospectivo realizado com pacientes maiores de 18 anos com fratura transtrocanteriana entre junho a dezembro de 2023. Foram coletados dados demográficos, clínicos e informações sobre o tempo de espera até a osteossíntese. As análises foram realizadas utilizando estatística descritiva e regressão logística.

**Resultados:**

A maioria dos pacientes (61,4%) eram do sexo feminino, com idade média de 74 anos. O tempo médio entre a fratura e o atendimento foi de 34 dias, e entre a fratura e a osteossíntese foi de 22,7 dias. Pacientes com comorbidades tiveram maior tempo de internação e complicações associadas. A taxa de mortalidade foi de 6,8%, com maior prevalência em mulheres idosas com comorbidades.

**Conclusões:**

O estudo demonstra que o tempo prolongado entre a fratura e a osteossíntese está associado a piores desfechos clínicos, especialmente em pacientes idosos com comorbidades. É fundamental otimizar o tempo de espera para cirurgia, visando à redução da morbimortalidade e melhor recuperação dos pacientes. ***Nível de Evidência IV; Estudo Observacional, Descritivo e Retrospectivo.***

## INTRODUCTION

A transtrochanteric fracture is a common type of fracture of the proximal femur, located between the greater trochanter and the lesser trochanter. This fracture is predominantly observed in the elderly, due to osteoporosis and the increased risk of falling in this age group.^1^ The transtrochanteric fracture represents one of the main causes of morbidity and mortality in elderly patients and requires a specific therapeutic approach to ensure the best possible recovery. In younger patients, these fractures may result from high-energy trauma, such as motor vehicle accidents.^2^

These fractures can vary in complexity, ranging from simple and stable to complex and unstable. The fracture pattern can be classified using the Evans classification system or the AO (*Arbeitsgemeinschaft für Osteosynthesefragen*) classification.^3^

The main risk factors are advanced age, osteoporosis, falls, and associated comorbidities.^4–7^ Diagnosis is made through a combination of medical history, physical examination, and imaging tests. X-rays are usually sufficient to confirm the fracture and assess its pattern. In complex cases or when other injuries are suspected, a computed tomography (CT) scan may be necessary.^8^

The treatment of transtrochanteric fractures can be conservative or surgical, depending on the type of fracture, the patient's clinical condition, and associated comorbidities. However, osteosynthesis is the treatment of choice in most cases due to its benefits in terms of recovery and early mobilization.^9^

Osteosynthesis, a surgical technique widely used in the treatment of bone fractures, plays a crucial role in the recovery of patients with transtrochanteric femur fractures.^1^ The main objectives of surgical management through osteosynthesis are fracture stabilization, pain relief, and early restoration of patient mobility.^3^ The choice of fixation method depends on several factors, including the fracture pattern, bone quality, and the patient's overall clinical condition.^4^ The most commonly used techniques include intramedullary nails and plates with screws, each with specific advantages in the context of transtrochanteric fracture treatment.^5^

This procedure presents both risks and benefits that the specialist and the patient should consider. The benefits include stable fracture fixation, allowing better alignment of bone fragments and facilitating the healing process; early mobilization of the patient, which is crucial for the prevention of secondary complications such as venous thromboembolism and pneumonia; it helps to significantly reduce associated pain, improving patient comfort and allowing better adherence to the rehabilitation protocol; it restores the function of the affected limb, allowing the patient to resume their daily activities and maintain independence; and it presents better clinical results in terms of functional recovery and patient quality of life.^1–5^

The main risks are infection at the surgical site, which may be superficial or deep, requiring additional treatment or, in severe cases, removal of the implant; possibility of failure of the implant used for fixation, either due to breakage or loosening, which may require further surgery; risk of perioperative complications, including cardiovascular events, venous thromboembolism, and pulmonary complications; in some cases, the fracture may not heal properly (nonunion) or may heal in an improper position (malunion), affecting function and requiring additional treatment; there is a risk of damage to soft tissues, blood vessels, and nerves adjacent to the fracture site, which may result in additional complications, and some patients may develop adverse reactions to the material used in the implants, such as allergies or inflammatory reactions.^6–10^

Therefore, this study aims to evaluate the epidemiological profile and waiting time for osteosynthesis in patients admitted due to transtrochanteric fractures at a tertiary public hospital in the Federal District.

## MATERIALS AND METHODS

A descriptive and retrospective observational study was conducted. The data collected are from patients over 18 years of age with transtrochanteric fractures who underwent osteosynthesis at the Regional Hospital of Taguatinga-DF between June and December 2023. This study was approved by the Human Research Ethics Committee of the Foundation for Teaching and Research in Health Sciences (FEPECS), under CAAE: 28246730 and opinion number 7,016,908. Signatures are being collected on the Free and Informed Consent Form.

Information was collected regarding the period of care, date of fracture, age, sex, associated comorbidities, type of fracture, mechanism of trauma, date of osteosynthesis, follow-up period, and outcome. To assess the epidemiological profile and waiting time for osteosynthesis in patients hospitalized due to transtrochanteric fractures, descriptive statistics were performed; to compare risk factors associated with waiting time and to assess the risk of mortality related to waiting time, logistic regression analysis was performed; to correlate the prevalence between sex and transtrochanteric fracture, the Chi-square test and Fisher's exact test were performed; to assess the risk of mortality related to waiting time and to assess the correlation between morbidity and mortality during the follow-up period, correlation analysis was performed. The analyses were performed using SPSS, version 22.

## RESULTS

During the period covered by this study, 98 patients with various fractures were admitted, including diaphyseal fractures of the femur: 19.4%; femoral neck fracture: 13.3% and other less frequent fractures: 22.4%, while transtrochanteric fractures accounted for 44.9%, of which 61.4% were female and 38.6% were male; participants had an average age of 74 years (range: 21–96 years) with a standard deviation (SD) of 17.7 years. The participants included in this study were mostly patients aged 60 years or older (90.9%), about 29.5% of patients denied any type of comorbidity, and in 54.5% of patients with some comorbidity, circulatory diseases (62.5%) and endocrine/nutritional/metabolic diseases (33.3%) were the most frequent. (Table 1)

**Table 1:** Sociodemographic data.

	Transtrocanteric				p-value
	Yes		No		
**Gender**					
Female	27	(61.4%)	19	(35.2%)	0.017
Male	17	(38.6%)	35	(64.8%)	
**Age group**					
**Average**	**74 years**		**52.5 years**		
18 to 19 years old	0	(0.0%)	1	(1.9%)	0.003
20 to 29 years old	1	(2.3%)	8	(14.8%)	
30 to 39 years old	1	(2.3%)	5	(9.3%)	
40 to 49 years old	2	(4.5%)	14	(25.9%)	
50 to 59 years old	6	(13.6%)	5	(9.3%)	
60 to 69 years old	1	(2.3%)	9	(16.7%)	
70 to 79 years old	11	(25.0%)	6	(11.1%)	
80 to 89 years old	14	(31.8%)	5	(9.3%)	
90 to 99 years	8	(18.2%)	1	(1.9%)	
**Comorbidities**					
Denies	13	(29.5%)	23	(42.6%)	0.172
Not available.	7	(15.9%)	8	(14.8%)	
Yes	24	(54.5%)	23	(42.6%)	
Circulatory System Diseases	15	(62.5%)	14	(60.9%)	
Endocrine, Nutritional, and Metabolic Diseases	8	(33.3%)	8	(34.8%)	
Nervous System Diseases	3	(12.5%)	5	(21.7%)	
Musculoskeletal and Connective Tissue Diseases	3	(12.5%)	0	(0.0%)	
Other less common comorbidities	11	(45.8%)	9	(39.1%)	

In 81.8% of cases, the most frequent mechanism of trauma was a fall from height or onto the ground. The average time between the occurrence of the fracture and treatment was 3.4 days (1–63 days) with a median of 10.1 days. The average period between fracture and osteosynthesis was 22.7 days (3–82 days) with a SD of 15.4 days (Table 2). In 25% of patients, outcomes were satisfactory, 40.9% were referred for rehabilitation, there were no unsatisfactory outcomes, and 6.8% of patients died; the average follow-up period for patients was 73.3 days (10–365 days) with a DP of 67.1 days. (Table 3)

**Table 2:** Data referring to care provided up to treatment.

	Transtrocanteric				p-value
	Yes		No		
**Time between the fracture occurring and my arrival.**					
**Average**	**3.4 days**		**1.8 days**		
1 to 2 days	39	(88.6%)	46	(85.2%)	0.016
3 to 9 days	2	(4.5%)	5	(9.3%)	
≥10 days	2	(4.5%)	1	(1.9%)	
Not available.	1	(2.3%)	2	(3.7%)	
**Mechanism of trauma**					
Fall height/to the ground	36	(81.8%)	20	(37.0%)	0.007
Other less frequent mechanisms	8	(18.2%)	34	(63.0%)	
**Period between fracture and osteosynthesis**					
**Average**	**22.7 days**		**25.1 days**		
1 to 2 days	0	(0.0%)	5	(9.3%)	0.018
3 to 9 days	6	(13.6%)	5	(9.3%)	
10 to 19 years old	16	(36.4%)	17	(31.5%)	
20 to 29 days	11	(25.0%)	10	(18.5%)	
30 to 39 days	7	(15.9%)	5	(9.3%)	
40 to 49 days	1	(2.3%)	4	(7.4%)	
≥ 50 days	2	(4.5%)	6	(11.1%)	
Not available.	1	(2.3%)	2	(3.7%)	

**Table 3:** Data regarding final outcome and follow-up of patients.

	Transtrocanteric				p-value
	Yes		No		
**Final outcome of the patient**					
Satisfactory	11	(25.0%)	7	(13.0%)	0.740
Rehabilitation	18	(40.9%)	25	(46.3%)	
Unsatisfactory	0	(0.0%)	4	(7.4%)	
Death	3	(6.8%)	2	(3.7%)	
Not available/not applicable	12	(27.3%)	16	(29.6%)	
**Follow-up period (in days)**					
**Average**	**73.2 days**		**96 days**		
< 30 days	4	(9.1%)	5	(9.3%)	0.173
30 to 60 days	19	(43.2%)	17	(31.5%)	
60 to 120 days	9	(20.5%)	11	(20.4%)	
>120 days	4	(9.1%)	11	(20.4%)	

The average time from injury to treatment for transtrochanteric fractures was twice as long as for other fractures at the institution (transtrochanteric; Yes: 3.4 days; no: 1.8 days), between the occurrence of the fracture and osteosynthesis, the average time was slightly shorter in patients with transtrochanteric fractures (Yes: 22.7 days; no: 25.1 days).

Regarding waiting times for care, surgical treatment, and follow-up with the outcome of patients with transtrochanteric fractures, patients who had a satisfactory outcome or went to rehabilitation had an average period between the occurrence of the fracture and care that was 0.4 and 0.8 days longer when compared to patients with other fractures. Regarding the average period between the occurrence of the fracture and osteosynthesis, in all outcomes, the average time in days was shorter when compared to other fractures; and during the follow-up period, patients with other fractures had considerably longer follow-up in all outcomes when compared to patients with transtrochanteric fractures. (Table 4)

**Table 4:** Average time in days from fracture occurrence to treatment, osteosynthesis, and follow-up of patients with transtrochanteric fractures.

	Transthoracic	
	Yes	No
**Time between fracture occurrence and care**		
Satisfactory	1.8	1.4
Rehabilitation	2.7	1.9
Unsatisfactory	-	1
Death	1	1
**Period between fracture and osteosynthesis**		
Satisfactory	19.2	33.2
Rehabilitation	23.8	26.6
Unsatisfactory	-	18.2
Death	18	25.5
**Follow-up period**		
Satisfactory	89	156
Rehabilitation	72	90
Unsatisfactory	-	150
Death	10	37

**Table 5:** Average time from initial treatment to osteosynthesis and follow-up of patients with associated comorbidities.

Period	Comorbidities			
	Denies	One	Two	Three or More
First contact	5.7 days	1.8 days	5.5 days	1.2 days
Performing osteosynthesis	28.2 days	17.8 days	21.6 days	21.8 days
Follow-up	83 days	86 days	45 days	62 days

Approximately 4 to 5 patients out of every 10 who underwent osteosynthesis at the Taguatinga Regional Hospital between June and December 2023 had transtrochanteric fractures, the majority of which were female (61.4%), with an average age of 73 years. All deaths from this type of fracture occurred in female patients aged between 68 and 88 years, all of whom had some type of comorbidity. These had an average period between fracture and treatment of one day, and between fracture and osteosynthesis of 21.7 days, with an average follow-up period of 24 days.

## DISCUSSION

The primary objective of the present study was to assess the epidemiological profile and waiting time for osteosynthesis in patients admitted due to transtrochanteric fractures at a regional hospital in the Federal District. It showed that women (61.4%) are more prone to transtrochanteric fractures. Furthermore, this type of fracture is more frequent in the elderly population (77.3%), with associated comorbidities (54.5%), mainly diseases of the circulatory system. These findings are consistent with those reported in the literature, which indicate a higher frequency of transtrochanteric fractures in the female population, the elderly, and individuals with more than one associated comorbidity.^11–15^ In addition, as evidenced in the present study, the presence of comorbidities related to the circulatory system has also been frequently reported in the literature, especially in elderly patients.^13,16^

The mechanism of trauma was mostly falls from height/to the ground in 81.8% of cases. These findings are consistent with the literature, which indicates that transtrochanteric fractures in the elderly are primarily caused by low-energy falls, with osteoporosis being a significant risk factor.^13,16^

The ideal time for performing osteosynthesis depends on several factors, including the type of fracture, the patient's condition, and the presence of complications, and can range from 24 to 72 hours. In general, the literature suggests that, in cases of polytrauma patients or patients with open fractures, fixation should occur early, within 24 hours, to minimize the risk of complications such as infections and fat embolism.^14,17^

In a study conducted by Mattisson L, et al. (2018), it was observed that patients who underwent surgery more than 36 hours after fracture had higher mortality rates at 30 days (9.8%) and 1 year (31%) compared to those who underwent surgery within 24 hours.^14^ Cruz V, et al. 2023 emphasize that surgeries should not exceed 48 hours, as this period contributes to reducing mortality in elderly patients with transtrochanteric fractures. The authors observed higher mortality when surgery was performed after 48 hours, reaching 38.5%, compared to 14.3% when surgery occurred within 24 hours.^17^ Patients admitted to Taguatinga Regional Hospital between June and December 2023 due to transtrochanteric fractures usually wait an average of 3.4 days between the occurrence of the fracture and receiving care, and up to 22.7 days for osteosynthesis. The data from this study demonstrate that patients with other types of fractures who underwent osteosynthesis during the same period tend to have a mean time between fracture occurrence and treatment that is twice as short (p-value 0.016), however, in relation to the average time until osteosynthesis, patients with transtrochanteric fractures take at least two days less than those with other fractures (p-value 0.018). Lu et al. (2022) emphasize that delays in surgical timing, especially in patients with comorbidities, can result in higher mortality rates after three years. The authors pointed out that factors such as advanced age and low albumin are independent predictors of long-term mortality after surgery for transtrochanteric fractures.^18^ Although patients with transtrochanteric fractures have a relatively shorter time to osteosynthesis compared to other fractures, the average time is still about eleven times longer than recommended (48 hours).^17^

This delay is associated with an increased risk of mortality, especially in patients with comorbidities. However, in the present study, even with surgery taking an average of 22.7 days, the frequency of deaths was lower than that observed in the literature (6.8%).^17^ However, patients with transtrochanteric fractures had twice the frequency of deaths when compared to other non-transtrochanteric fractures, all of whom were female, aged between 68 and 88 years, and had some type of comorbidity. This finding is consistent with the literature, which highlights that older patients with comorbidities are at greater risk of complications and mortality when surgery is postponed.^11–14,17^

In the present study, patients with one comorbidity had an average of 1.8 days until the first visit, 17.8 days until osteosynthesis, and 86 days of follow-up; those with two comorbidities had 5.5 days until the first visit, 22 days until osteosynthesis, and 45 days of follow-up; and patients with three or more comorbidities had an average of 1.2 days until the first visit, 21.8 days until osteosynthesis, and 62 days of follow-up. However, when evaluating patients without any comorbidities, it was found that the first consultation took up to 6 days, osteosynthesis required 28 days, and follow-up lasted 83 days. This demonstrates that having an associated comorbidity was not a factor that influenced the waiting time for the first consultation and osteosynthesis at the institution.

However, it should be noted that the literature has observed that the presence of preoperative comorbidities significantly influences the waiting time for surgery. In patients with three or more comorbidities, the preoperative waiting time is usually longer, contributing to an increased risk of deep vein thrombosis (DVT).^13^ Cruz V, et al. (2023) also highlight that delays in surgery can be influenced by factors such as the need to stabilize the patient, hospital bureaucracy, and lack of available resources.^17^

Another factor that plays a key role in recovery and the development of complications is the length of hospital stay. Patients with transtrochanteric fractures often require longer hospital stays due to the complexity of the fracture and prolonged recovery. In the study by Mattisson et al. (2018), it was observed that the length of hospital stay was directly related to the risk of postoperative complications, especially in older patients with multiple comorbidities.^14^

The incidence of deaths related to all cases of transtrochanteric fractures in the study period was 0.7. However, when evaluating only female patients, it was observed that for every ten cases of transtrochanteric fractures, one case could result in death, mainly in cases of patients aged 70 years or older and with three or more associated comorbidities.

Lu Y, et al. (2022), in their study aimed at investigating risk factors associated with mortality three years after surgery for intertrochanteric fractures in the elderly, demonstrated that in this population, cumulative mortality was 9.6% in the first year, 16.7% in the second year, and 24.4% in the third year after surgery. The greatest risk group is elderly individuals with low albumin levels.^18^ Li X, et al. (2021) also highlighted a cumulative mortality of 10.8% during the follow-up period, with an annual mortality rate of 5.4%. These findings are closer to those of the present study.^12^

Overall, this study highlights that during the period studied, there was a high prevalence of elderly women and a significant waiting time for surgical treatment. This delay is directly associated with an increased risk of complications and mortality, especially in patients with comorbidities. In addition, prolonged hospitalization and inadequate follow-up also contribute to adverse long-term outcomes.

In this scenario, despite overall satisfaction in 25% of patients with transtrochanteric fractures who underwent osteosynthesis between June and December 2023 at the Taguatinga Regional Hospital. It remains essential to improve clinical outcomes for these patients by reducing waiting times for osteosynthesis, implementing effective strategies to manage comorbidities, and ensuring comprehensive long-term follow-up in the postoperative period. These efforts may contribute to reducing the frequency of morbidity and mortality associated with transtrochanteric fractures and improving the quality of life of affected patients.

## CONCLUSION

After conducting this study, we conclude that the epidemiological profile of patients with transtrochanteric fractures in a public hospital in the Federal District is mainly composed of elderly individuals, predominantly female, with some associated comorbidity. The waiting time between fracture and osteosynthesis was longer than recommended, with an average of 22.7 days, which may negatively impact clinical outcomes, especially in patients with comorbidities. Although the study did not find a direct relationship between the number of comorbidities and the waiting time for care or surgery, the literature provides evidence that prolonged waiting times are associated with a higher risk of complications, including mortality and rehabilitation difficulties. Finally, we emphasize the importance of optimizing waiting times for surgery in transtrochanteric fractures, aiming to improve outcomes and reduce morbidity and mortality, especially in a high-risk population such as the one studied.
